# High-Field Functional Imaging of Pitch Processing in Auditory Cortex of the Cat

**DOI:** 10.1371/journal.pone.0134362

**Published:** 2015-07-30

**Authors:** Blake E. Butler, Amee J. Hall, Stephen G. Lomber

**Affiliations:** 1 Department of Physiology and Pharmacology, University of Western Ontario, London, Ontario, Canada; 2 Brain and Mind Institute, University of Western Ontario, London, Ontario, Canada; 3 Department of Anatomy and Cell Biology, University of Western Ontario, London, Ontario, Canada; 4 Department of Psychology, University of Western Ontario, London, Ontario, Canada; 5 National Centre for Audiology, University of Western Ontario, London, Ontario, Canada; Kyoto University, JAPAN

## Abstract

The perception of pitch is a widely studied and hotly debated topic in human hearing. Many of these studies combine functional imaging techniques with stimuli designed to disambiguate the percept of pitch from frequency information present in the stimulus. While useful in identifying potential “pitch centres” in cortex, the existence of truly pitch-responsive neurons requires single neuron-level measures that can only be undertaken in animal models. While a number of animals have been shown to be sensitive to pitch, few studies have addressed the location of cortical generators of pitch percepts in non-human models. The current study uses high-field functional magnetic resonance imaging (fMRI) of the feline brain in an attempt to identify regions of cortex that show increased activity in response to pitch-evoking stimuli. Cats were presented with iterated rippled noise (IRN) stimuli, narrowband noise stimuli with the same spectral profile but no perceivable pitch, and a processed IRN stimulus in which phase components were randomized to preserve slowly changing modulations in the absence of pitch (IRNo). Pitch-related activity was not observed to occur in either primary auditory cortex (A1) or the anterior auditory field (AAF) which comprise the core auditory cortex in cats. Rather, cortical areas surrounding the posterior ectosylvian sulcus responded preferentially to the IRN stimulus when compared to narrowband noise, with group analyses revealing bilateral activity centred in the posterior auditory field (PAF). This study demonstrates that fMRI is useful for identifying pitch-related processing in cat cortex, and identifies cortical areas that warrant further investigation. Moreover, we have taken the first steps in identifying a useful animal model for the study of pitch perception.

## Introduction

Pitch is the perceptual correlate of sound frequency, and the way that pitch changes over time defines the melody of a musical piece. In addition, verbal pitch is fundamental to language acquisition both for tonal languages, where pitch variations provide basic lexical information, and for non-tonal languages where pitch is used to convey emotional and other paralinguistic information. Moreover, pitch cues are critical for extracting meaningful signals from a background of competing auditory information, such as is encountered in the classic “cocktail party” phenomenon (See [1] for a review). Thus, the accurate encoding of pitch is critical to establishing faithful representations of biologically relevant acoustic stimuli in the listener’s environment.

The vast majority of pitch-evoking sounds present in the environment contain energy at a fundamental frequency and at integer multiples of that frequency, known as harmonics. However, through complex spectrotemporal processing, a singular pitch is perceived that corresponds to the fundamental of the complex source. This pitch percept is the result of two pitch codes that are established in parallel at the level of the cochlea: a spectral code based on the site of maximal displacement along the tonotopically organized basilar membrane, and a temporal code based on the rate of action potential generation in afferent neurons that synapse at that characteristic place. While these cues are encoded at the auditory periphery, a growing literature suggests that the percept of pitch is first established cortically, at a point beyond primary auditory cortex (A1). This is despite evidence that orthogonal tonotopic and periodotopic maps exist in A1 that may provide the capacity for pitch extraction at this level [2–3]. Still, studies using electroencephalography (EEG) or functional magnetic resonance imaging (fMRI) in humans suggest that pitch processing is localized either to an area along lateral Heschl’s gyrus [4–7] or planum temporale [8–11]. These results have been supported by electrophysiological recordings in non-human primates that fail to find pitch-sensitive neurons in A1, instead localizing pitch processing to a cortical belt region along the anterolateral border of A1 in the marmoset [12] and to the lateral belt region in the macaque [13].

While this convergence of evidence suggests pitch emerges beyond primary auditory cortex, how the peripherally-encoded pitch cues are converted into a singular percept remains poorly understood. This represents a critical step in object representation, as perceptual judgements and consequent actions are not based on individual analysis of the frequency components encoded by peripheral structures, but instead occur following higher-level pitch perception. For example, while two speakers may produce energy at the same frequency components, the way in which the sum of these components is represented by pitch-responsive neurons helps to discriminate the speakers and may affect the meaning extracted from speech. The use of animal models allows for the combination of traditional fMRI studies of pitch perception with more invasive methods that will provide the necessary information to address this open question. Still, the neural underpinnings of pitch perception have yet to be addressed in a number of traditional models of auditory function. For example, the ability of cats to perceive the pitch of complex auditory stimuli has long been known [14–15], yet the cortical region subserving this function remains unidentified. Using fMRI, the current study seeks to identify areas of cat auditory cortex that contribute to pitch perception and aims to provide a basis for future studies to further examine the topic of pitch encoding in the cat. A better understanding of pitch perception will provide insight into how listeners engage with meaningful stimuli in their environment. Furthermore, it may help elucidate issues related to impoverished pitch processing, such as those reported by individuals with congenital amusia [16], and may provide a basis for improving the encoding of pitch by cochlear implant users.

## Materials and Methods

### Animal Preparation

**Fig 1:**
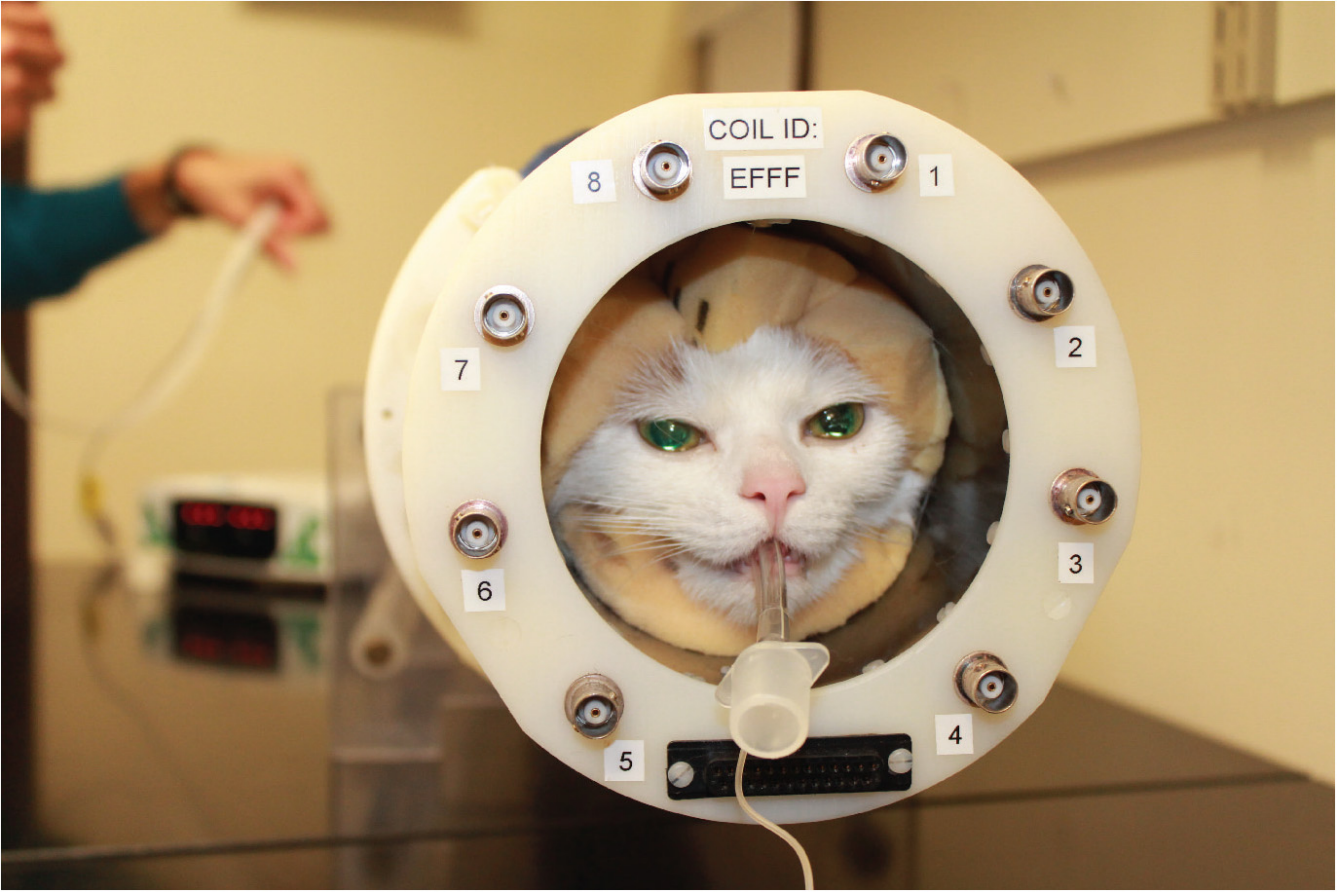
An anesthetized animal in the 8-channel RF coil. The head is surrounded by foam that serves to minimize movement and attenuate scanner noise. Also pictured is the intubation tube which permits the administration of isoflurane anesthesia.

Ten adult (>6 months) domestic cats (Liberty Labs, Waverly, NY) participated in this experiment, and were housed as a clowder. All procedures were approved by the University of Western Ontario’s Animal Use Subcommittee of the University Council on Animal Care and were in accordance with the guidelines specified by the Canadian Council on Animal Care [17]. Normal hearing thresholds were verified for each animal using click-evoked auditory brainstem responses (ABRs) measured with an Eclipse EP15 Diagnostic ABR system (Interacoustics A/S, Denmark). Prior to each imaging session, cats were pre-medicated with a mixture of atropine (0.02 mg/kg s.c.) and acepromazine (0.02 mg/kg s.c.). This combination has been shown to reduce the amount of general anesthesia necessary during scanning [18], thus minimizing anesthetic-induced cortical suppression. Each animal was anaesthetized 20 minutes later with a cocktail of ketamine (4 mg/kg i.m.) and dexdomitor (0.025 mg/kg i.m.). Pilot studies in our lab comparing anesthetic regimes for functional imaging in the cat have identified this as the ideal combination of drugs to maintain adequate sedation for the duration of a scanning session while optimizing BOLD response. This procedure, described in length by Brown and colleagues [19] has been shown to be effective in demonstrating stimulus-evoked activity in the cat’s auditory cortex [19–20]. Once anesthetized, the animal was intubated and an indwelling catheter was placed in the saphenous vein to facilitate the maintenance of anesthesia. Once prepared, the animal was placed in a sternal position within a custom-built Plexiglas apparatus (Fig 1). Body temperature was maintained with heating discs placed inside the apparatus, and vitals including respiratory rate, end-tidal CO_2,_ heart rate, non-invasive blood pressure, and blood-oxygen saturation were continuously monitored. MRI-compatible earphone inserts comprised of sound-attenuating foam surrounding a tube designed to deliver sound stimuli as closely as possible to the tympanic membrane were placed in the ear canals. The animals head was then placed inside a custom-built 8-channel radio frequency (RF) coil, and stabilized with sound dampening compression foam padding that further attenuated scanner noise. Finally, the animal and the Plexiglas apparatus were placed inside the bore of the magnet. For the remainder of the session, anesthesia was maintained through continuous administration of ketamine (0.6–0.75 mg/kg/hr i.v.) and spontaneous inhalation of isoflurane (0.4–0.5%). Following each scanning session, anesthesia was discontinued and animals were monitored until they recovered from anesthetic effects. The intubation tube was removed when the cat exhibited a gag reflex and increased jaw tone. Following successful recovery, the indwelling catheter was removed and cats were returned to their clowder. Every effort was made to minimize animal trauma during the entire scanning period.

### Stimuli

All stimuli were generated in MatLab (MathWorks) and presented at 85 dB SPL using in-house custom software (Microsoft Visual Studio) on a Dell laptop through an external Roland Corporation soundcard (24-bit/96kHz; Model UA-25EX) and Pyle Pro external amplifier (Model PCAU11) to MRI-compatible foam earphone inserts (Sensimetric Model S14). The narrowband Gaussian noise (NBN) stimulus was 400 ms in duration and covered a ¼ octave band centered at 10 kHz (48 dB/octave band pass filter, 10 ms linear onset/offset ramps). The IRN stimulus was generated by first creating a 1s Gaussian noise. This noise sample was then duplicated and added to itself following a 2.5ms delay. This delay-and-add process was repeated 16 times to create a stimulus with a perceivable pitch of 400 Hz. This process resulted in a 1040 ms stimulus that increased in amplitude gradually over the first 40 ms and decreased gradually over the last 40 ms. To correct for this, the first and last 320 ms were removed to create a 400 ms stimulus with equal amplitude across its entire duration. Finally, the IRN stimulus was band pass filtered to match the spectral range of the narrowband noise stimulus (a ¼ octave band centered at 10 kHz, 48 dB/octave filter) and 10 ms onset and offset ramps were applied. In addition, an IRNo stimulus was generated as described by Barker and colleagues [11]. In short, the IRN stimulus described above was sampled with a rectangular window with duration equal to the IRN delay (2.5 ms). A fast Fourier transform (FFT) was used to determine the phase and amplitude spectra of the sample and phase components were randomized. An inverse FFT was then used to regenerate the time representation. The analysis window was advanced by 2.5 ms and this process was repeated through the duration of the stimulus. The regenerated samples were concatenated (preserving onset times) to create a 400 ms IRNo stimulus with fine structure information (and resulting pitch percept) removed. The IRNo stimulus was band pass filtered to match the spectral range of the IRN and narrowband noise stimuli, and 10 ms linear onset and offset ramps were applied. This limited bandwidth minimized interference between the stimuli used in the current study and scanner noise, which is largest at lower frequencies, while ensuring stimuli were well within the normal range of feline hearing. Moreover, the pitch percept evoked by IRN stimuli is dependent upon temporal repetition rather than resolvable spectral content such that traditional spectral and temporal envelope models of pitch perception are unable to explain the perception of IRN pitch [21]. Thus, high-pass filtering has a minimal impact on the pitch percept of IRN stimuli. Each of the stimulus types (NBN, IRN, IRNo) were presented in bursts consisting of 30 repetitions of the 400 ms stimulus with a 100 ms interstimulus interval (ISI), for a total block duration of 15 s.

### MRI Parameters & Scanning Protocol

All data were acquired on an actively shielded 68 cm 7 T horizontal bore scanner with a DirectDrive console (Agilent, Santa Clara, California) equipped with a Siemens AC84 gradient subsystem (Erlangen, Germany) located at the Robarts Research Institute, and operating at a slew rate of 300 mT/m/s. An in-house designed and manufactured conformal 10 cm cylindrical 8-channel transceive radiofrequency coil was used for all imaging sessions. Magnetic field optimization (B_0_ shimming) was performed using an automated 3D mapping procedure [22] over the specific imaging volume of interest. For each cat, functional volumes were collected over the whole brain in an axial orientation using a single shot echo-planar imaging (EPI) acquisition with grappa acceleration (R = 3) and the following parameters: TR = 2000 ms; TE = 19 ms; flip = 70 degrees; slices = 26 x 1 mm; matrix = 96 x 96; FOV = 84 x 84 mm; acquisition voxel size = 0.88 mm x 0.88 mm x 1.0 mm; acquisition time = 2 s/volume; BW = 3719 Hz/px. Images were corrected for physiological fluctuations using navigator echo correction [23]. A high-resolution anatomical reference volume was collected along the same orientation and FOV as the functional images using a FLASH imaging sequence (TR = 750 ms; TE = 8ms; matrix = 256 x 256; acquisition voxel size = 281 μm x 281 μm x 1 mm). At the start of each scanning session, a broadband noise stimulus (0–25 kHz, 400 ms bursts with 10 ms onset/offset ramps, 100 ms ISI) was presented in an ON-OFF block design to confirm acoustically-evoked activity in auditory cortex. Following this confirmation, each of the stimuli were presented in ON-OFF continuous block protocols with each block consisting of 15 volumes (TR and TA = 2s). Three to six blocks of each stimulus type were collected in a scanning session. While the choice of a continuous scanning protocol may have a minimal impact on the salience of pitch-evoking stimuli, previous work in our lab has suggested that continuous scanning optimizes the quality and quantity of data obtained in the anesthetized cat [20]. Moreover, tonotopic map representation in human auditory cortex has been shown to be only minimally distorted by scanner noise [24]. Three blocks of each stimulus type were collected in a run, and stimulus blocks were interleaved with baseline blocks during which no stimulus was presented.

### Data Modelling

#### Individual animal analyses

Functional image volumes from each animal were analyzed separately using SPM8 (Wellcome Trust Centre for Neuroimaging, UCL, London, UK) and MatLab software. Image volumes for each animal were reoriented, realigned to the mean, motion corrected, co-registered to the high-resolution structural image from the same session, and smoothed with a 2 mm Gaussian full width at half maximum (FWHM) kernel. Some animals were scanned on multiple days; in these cases, functional volumes were co-registered to the anatomical image collected on the first day of scanning to facilitate data analysis across scanning sessions. A general linear model of the functional data was created for each animal imaged. Head motion parameters (3 translational and 3 rotational measures) were included as regressors of no interest in the model to account for movement-related artifacts. Following model estimation, t-contrasts were generated for each of the stimuli presented (stimulus–baseline) as well as for the difference in activation between the IRN and NBN stimuli (IRN–NBN) and for the difference between IRN and IRNo stimuli (IRN–IRNo). Hand drawn region of interest (ROI) masks were generated for the auditory cortex of each animal based on anatomy.

#### Group-level analysis

Group-level analysis was performed using SPM8 (Wellcome Trust Centre for Neuroimaging, UCL, London, UK) and MatLab software. Image volumes from all animals were reoriented, realigned to the mean, co-registered to the high-resolution structural image from the same session, normalized to an in-house generated feline anatomical template, and smoothed with a 2 mm Gaussian full width at half maximum (FWHM) kernel. Full details regarding the generation of the feline template will be addressed in a future manuscript; in short, 12 anatomical scans from a 7T high-field MRI scanner were preprocessed with SPM8 (Wellcome Trust Centre for Neuroimaging, UCL, London) and MatLab to align them to a common coordinate system. The images were then normalized and averaged first to a reference image, then to the average generated by first pass analysis. The resultant second pass average was then smoothed and provided for group analyses. A general linear model of the normalized functional data was created with head motion parameters (3 translational and 3 rotational measures) included as regressors of no interest. Following model estimation, t-contrasts were generated for each of the stimuli presented (stimulus–baseline) as well as for the difference in activation between the IRN and NBN stimuli (IRN–NBN) and for the difference between IRN and IRNo stimuli (IRN–IRNo).

### BOLD Signal Representations and Time Courses

Data from each animal were first extracted separately, followed by group-level extraction. In each case, a voxelwise threshold of p<0.001 (uncorrected) and a cluster-level threshold of p<0.05 (FWE-corrected) were applied to all results. Within each cluster, peak voxels showing a greater BOLD signal in response to IRN stimuli than either the NBN or IRNo stimuli were assigned to one of 13 defined areas of cat auditory cortex using a method described previously [19,25]. Briefly, the co-ordinates of a voxel of interest (VOI) were recorded from the output of the linear model, and plotted in three-dimensional space on the high-resolution anatomical scan for an individual animal, or on the anatomical template in the case of group analysis. Landmarks previously shown to delineate cortical areas were then used to determine the location of the BOLD activity peak. The mean percent signal change (PSC) at each voxel within a cluster was computed across the entire block of each stimulus type and normalized by subtracting the mean BOLD signal during baseline blocks. To visualize activity related to pitch processing, the difference in mean BOLD activity was then computed for each voxel (IRN-NBN and IRN-IRNo), and plotted on anatomical images. Additionally, mean PSCs across voxels comprising a 1 mm sphere surrounding each VOI were computed for each of the 15 volumes in a stimulus block individually to examine the time course of BOLD signal changes for each stimulus type and contrast of interest. One-sample t-tests corrected for multiple comparisons were used to determine where BOLD signals differed significantly from baseline. Related-samples t-tests corrected for multiple comparisons were used to compare the PSC elicited by different stimulus types.

## Results

**Fig 2:**
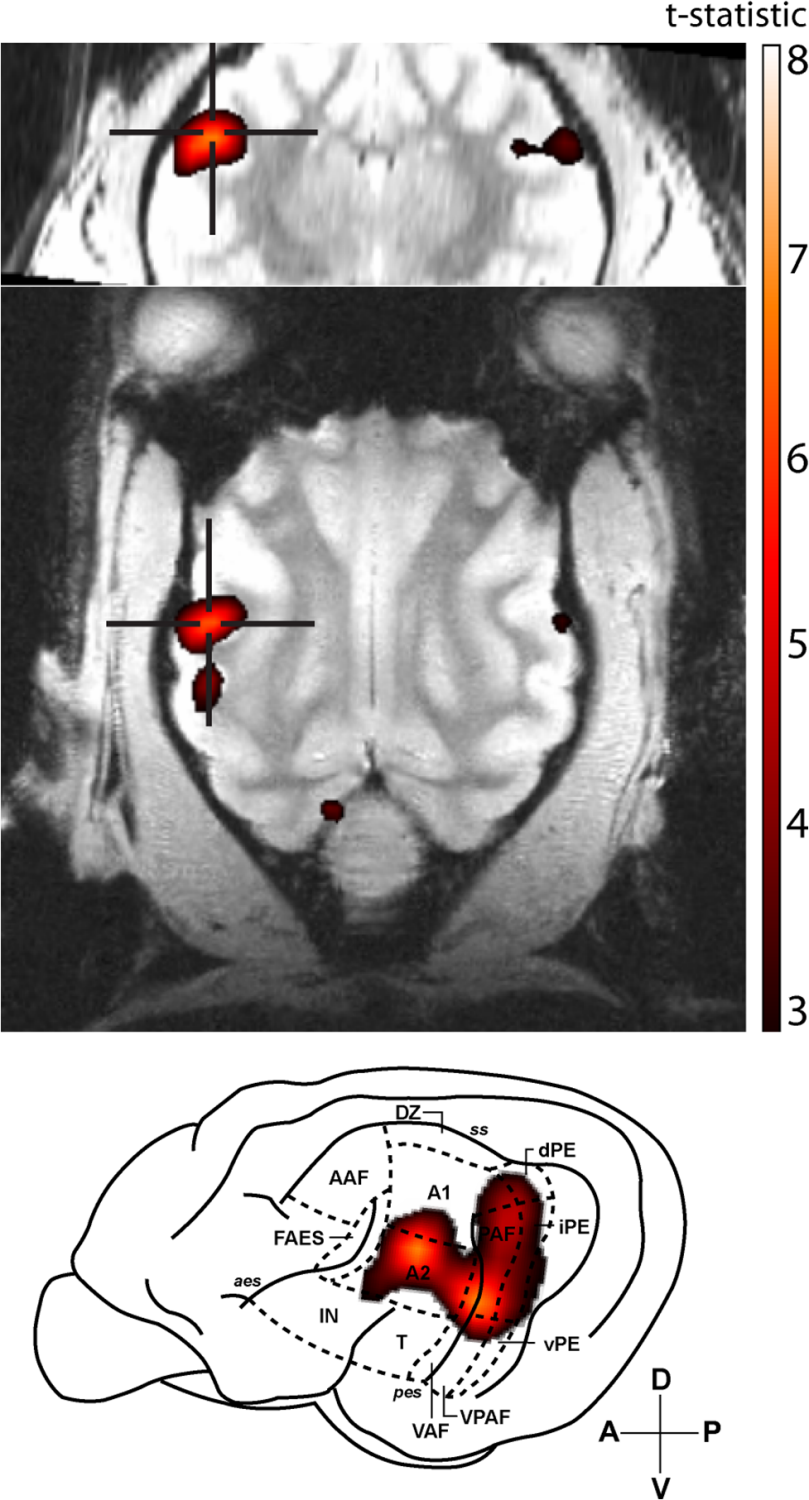
Representative T-statistic map. BOLD activity for the IRN-NBN contrast in a single animal. Significant activity was observed across multiple fields of the auditory cortex, with significant peaks in both the posterior auditory field (PAF) and second auditory cortex (A2). Maps are overlaid on coronal (top row) and horizontal (middle row) anatomical slices, and are superimposed on a lateral view of cat cortex. The local peak signal change maxima are indicated by crosshairs. A: anterior, P: posterior, V: ventral, D: dorsal.

In order to determine the area of cat auditory cortex that selectively responds to stimuli with pitch, an IRN stimulus was contrasted against a narrowband noise stimulus that consisted of identical spectral content, but which did not elicit a pitch percept. Five of the ten animals scanned showed clusters of significant activity for this contrast. At an individual-animal level, these clusters of significant BOLD activity encompassed multiple areas of the auditory cortex (an example is shown in Fig 2) with peak levels of activity typically observed in areas occupying the banks of the posterior ectosylvian sulcus, including the posterior auditory field (PAF), ventral auditory field (VAF), and ventral posterior auditory field (VPAF).

**Fig 3:**
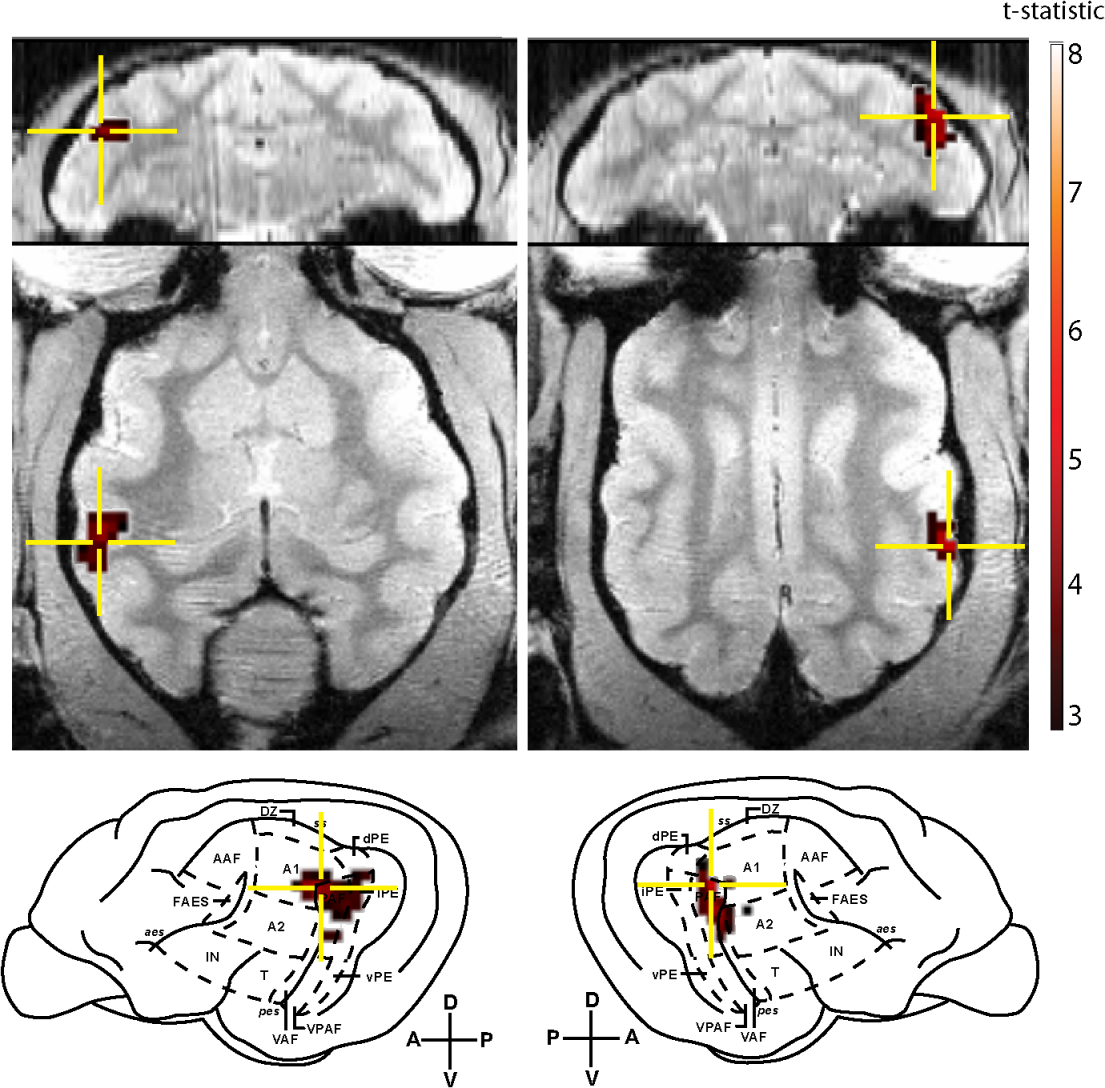
Group T-statistic maps. BOLD activity for the IRN-NBN contrast in the group of cats tested. The peaks of significant clusters of activity were localized to voxels within the posterior auditory field (PAF) bilaterally. Maps are overlaid on coronal (top row) and horizontal (middle row) anatomical slices, and are superimposed on a lateral view of cat cortex. The local peak signal change maxima are indicated by crosshairs. A: anterior, P: posterior, V: ventral, D: dorsal.

**Fig 4:**
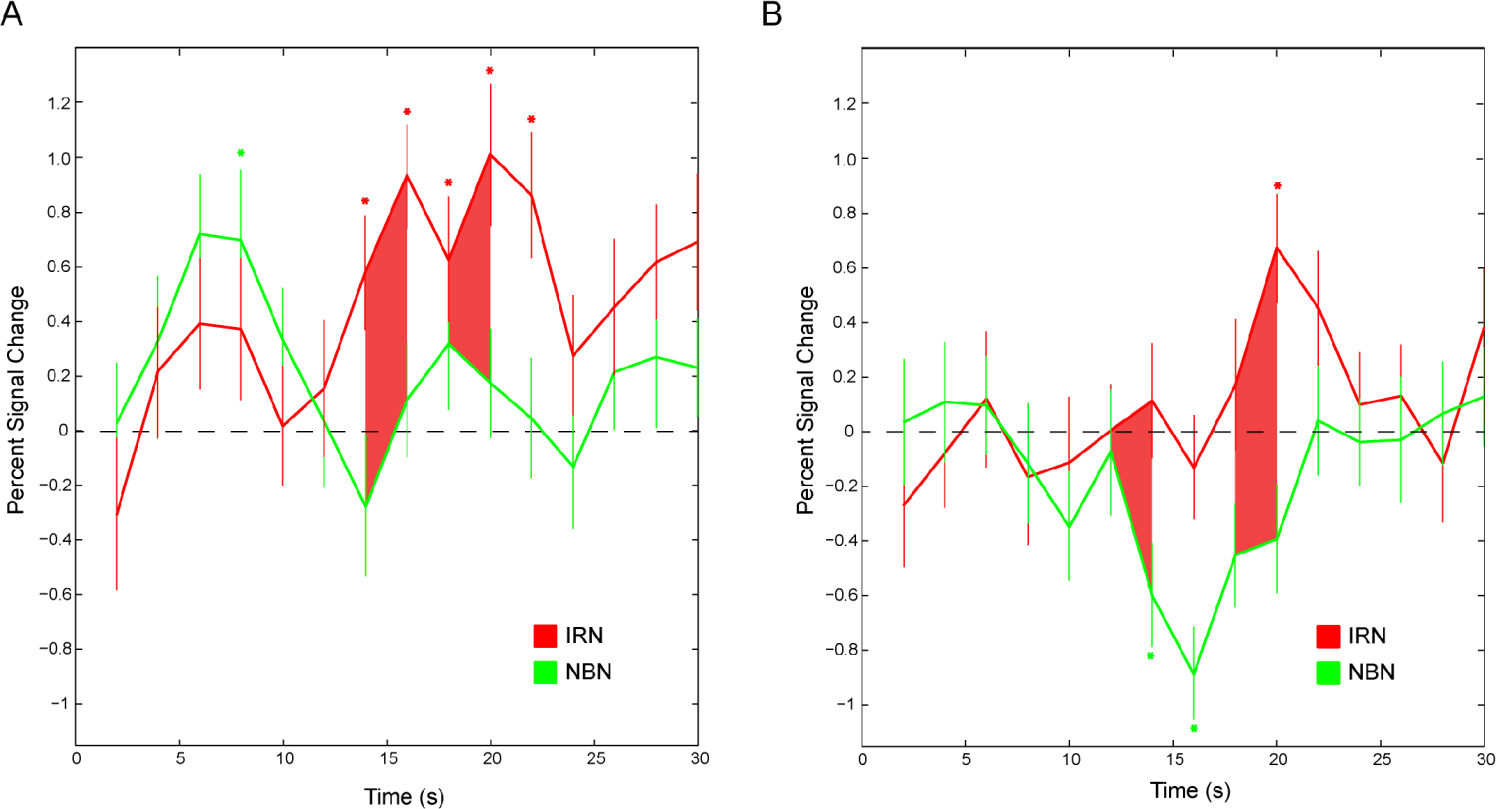
Hemodynamic time courses for bilateral PAF activity averaged across animals. The mean percent signal change in voxels within a 1mm sphere of the peak voxels are plotted for each volume in response to IRN (red) and NBN (green) stimuli. Error bars indicate the standard error of the mean. Points where an individual time course differs significantly from zero are marked by an asterisk while points where the two time courses significantly differ from each other are indicated by shading.

A group-level analysis was also performed to identify the locus of pitch processing across animals. The IRN-NBN contrast revealed large, significant clusters of BOLD activity centered bilaterally over PAF (Fig 3). The extracted BOLD signal time course for a 1 mm sphere surrounding the maximally significant voxel for this contrast are presented in Fig 4 for the left (panel A) and right (panel B) hemispheres. While a majority of pitch imaging studies in humans have employed this contrast (IRN-NBN), it has been suggested that this contrast is insufficient to truly image pitch-specific regions since the process involved in generating IRN stimuli introduces modulations of signal strength that are unrelated to the pitch percept, and which are not present in filtered Gaussian noise [11]. In an attempt to further isolate pitch-related information, BOLD signal changes in response to an IRN stimulus were contrasted against the response to a no-pitch IRN stimulus (IRNo) in which phase components are randomized to remove the pitch percept, but preserve the slowly changing signal modulations. This contrast (IRN-IRNo) revealed some auditory cortex activity bilaterally, but this failed to reach significance (data not shown).

## Discussion

### Pitch-related activity is clustered about pes

**Fig 5:**
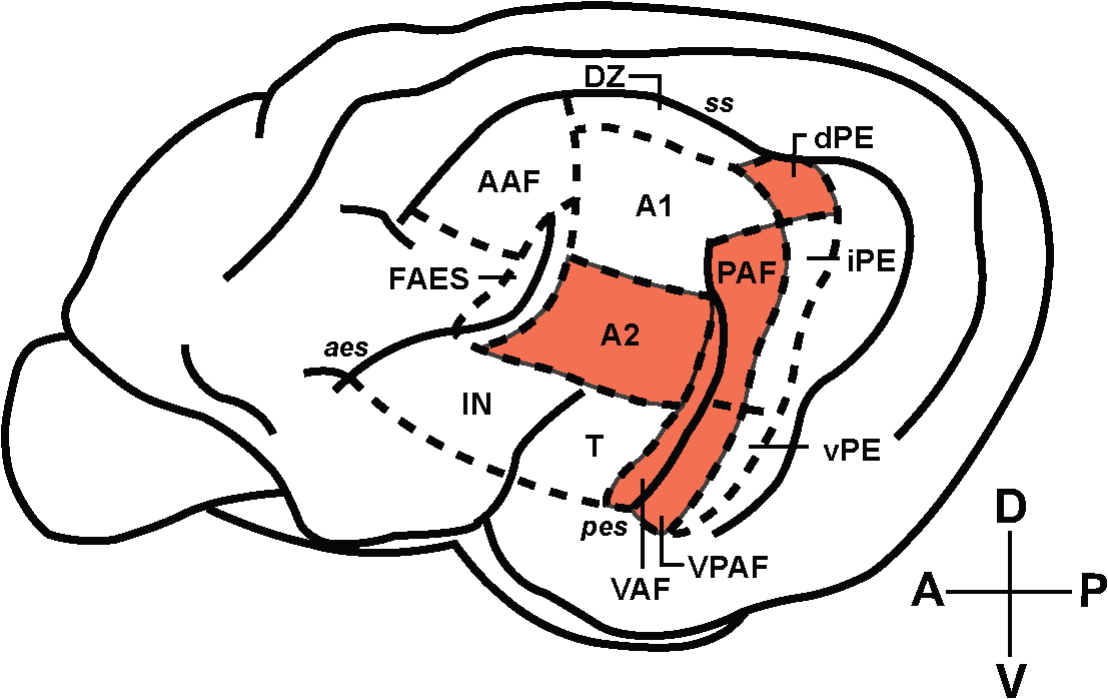
Summary of the areas of cat auditory cortex that respond to pitch-evoking stimuli at the individual-animal level. Individual-level analyses revealed clusters of pitch-related BOLD activity (IRN-NBN contrast) that spread across a number of areas of auditory cortex. Shading marks areas where significant peaks of BOLD activity were located in at least one animal.

Single-animal based analysis of pitch perception revealed large clusters of BOLD activity that encompassed a number of fields of the cat auditory cortex, primarily those surrounding the posterior ectosylvian sulcus (see Fig 5 for a summary). This area has previously been considered as a candidate for processing auditory patterns in the spectral and temporal domains, such as those found in communicative signals [26]. Group-level analysis revealed that pitch-related BOLD activity is centred over the posterior auditory field (PAF). The presence of pitch-responsive neurons in PAF satisfies a number of a priori hypotheses based on human and animal studies. Maximal pitch-related activation was not observed to occur in either primary auditory cortex (A1) or the anterior auditory field (AAF), which comprise the core auditory cortex in the cat [27]. Rather, it was localized to an area of auditory cortex that is functionally upstream from A1/AAF that has been shown to receive direct projections from core areas [27]. Interestingly, the cluster of activity in the left hemisphere *did* extend over the portion of A1 that a previous optical imaging study [2] determined corresponds to a periodicity of 400 Hz (the pitch elicited by the IRN stimulus in the current study). The locus of maximal activity was the dorsal aspect of PAF, which is located adjacent to the low-frequency border of A1, providing analogous homology to that observed in the marmoset [12]. While the IRN-NBN contrast revealed strong, bilateral activation of PAF, the IRN-IRNo contrast failed to reveal any significant activity.

### Frequency- and pitch-tuning in PAF

Because pitch is considered to be the functional correlate of stimulus frequency, one would expect to find a frequency-based representation in pitch-responsive cortex. Indeed, each of areas PAF has been previously shown to display tonotopic organization [28–29]. In addition, neurons within a pitch-responsive area would be expected to respond to a variety of stimuli that evoke a common pitch (e.g. to a 1 kHz pure tone as well as a stimulus that evokes a 1 kHz percept in the absence of energy at the fundamental frequency). While early studies suggested that PAF neurons showed narrow, single-frequency tuning curves [26,30], extracellular single-unit recordings have identified a significant proportion of PAF neurons with broader, multipeaked tuning curves shaped by complex patterns of inhibition [31–32]. Loftus and Sutter [32] also identified PAF neurons that produce long-latency responses to tonal stimuli that are unrelated to stimulus offset. These late responses might contribute to spectral integration across multiple frequencies, or to the convergence of information from parallel pathways such as those involved in spectral/temporal pitch coding. PAF has repeatedly been shown to be critical to sound localization [33–35]. Thus, one possible role of pitch-sensitive neurons in PAF involves the interpretation of pitch-based localization cues resulting from head-related transfer functions; these cues help to overcome the cone of confusion that remains after interaural differences are computed (see [36] for review).

In PAF, differences in activity between pitch-evoking and noise stimuli involved a negative BOLD signal in response to narrowband noise in addition to an accompanying increase in BOLD signal in response to IRN. The BOLD signal is a measure of the ratio of oxygenated to deoxygenated blood and is an indirect measure of neural activity; as such the meaning of a negative BOLD signal (relative to baseline) is not immediately evident. However, a growing body of research suggests that a negative BOLD signal reflects GABA-mediated [37] inhibitory signalling and concurrent arteriolar vasoconstriction across cortical areas [38–40]. Thus, the response to pitch in much of *pes*-adjacent cortex appears to reflect a combined sensitivity to pitch-evoking stimuli and suppression of auditory stimuli without pitch.

### Pitch-evoked vs. spectrotemporal modulation-evoked activity

Many human studies of pitch perception combine minimally invasive functional imaging techniques with IRN stimuli that target temporal cues to elicit the perception of pitch. In the majority of these studies, activity elicited in response to IRN is contrasted against a spectrally-matched noise stimulus to isolate pitch-related activity [5,41–42]. However, it has been suggested that slowly-varying spectrotemporal modulations present in IRN stimuli, but which are unrelated to stimulus pitch may confound interpretation of the IRN-NBN contrast [11]. This is despite evidence that cortical areas identified by the IRN-NBN contrast in humans are also activated by a wide variety of pitch-evoking stimuli that do not contain similar slowly varying modulation [6]. To further examine this issue, an IRNo stimulus was created which contained slow spectrotemporal modulations in the absence of pitch. A contrast between the pitch-evoking IRN stimulus and the IRNo stimulus did not reveal significant activity at the group level. A lower level of activity is congruent with the findings of Barker and colleagues [11] who also observed a marked decrease in the number of subjects showing significant differences in BOLD activity between the two stimulus types (10/16 subjects in their IRN-NBN contrast vs. 3/16 for their IRN-IRNo contrast). Moreover, the possibility that some animals included in the current group analysis may have had partially supressed cortical activity due to anesthetic effects may also have led to a more conservative estimate.

### Making a case for PAF as the centre of pitch processing

While there remains some debate regarding the existence of a “pitch centre”, there are agreed upon criteria that should be satisfied by a candidate area. For example, the area should generalize across pitch-evoking stimuli and should demonstrate a pattern of responses that correlates with the pitch salience. Thus, future studies should employ a wider variety of pitch-evoking stimuli to determine whether any of the candidate areas identified in the current study satisfy these criteria. In the current study five of ten animals scanned showed pitch-related activity that reached significance for one of the contrasts performed. This could be attributed to a number of factors including anaesthetic effects on cortical activity, as well as the reduced pitch salience of IRN stimuli relative to common pitch-evoking stimuli such as those used for communication. Indeed a more robust examination outlining the role of pitch salience would help to address this concern. Finally, the spatial resolution of the current study does not allow for the exclusion of pitch processing at the individual neuron level in areas lower in the auditory hierarchy. Thus, single-cell electrophysiology should be undertaken in an attempt to identify pitch-sensitive neurons in PAF, and to further support the absence of such neurons in primary auditory fields. Localizing pitch-responsive neurons in cat auditory cortex will provide a flexible model in which to examine pitch processing, as well as changes that may occur in response to deprivation and/or the introduction of auditory prostheses.
